# Differences in Osseous Shoulder Morphology, Scapulothoracic Orientation, and Muscle Volume in Patients With Constitutional Static Posterior Shoulder Instability (Type C1) Compared With Healthy Controls

**DOI:** 10.1177/03635465241233706

**Published:** 2024-03-15

**Authors:** Doruk Akgün, Henry Gebauer, Alp Paksoy, Frederik Schafer, Eva Herbst, Daniel Karczewski, Marc-Frederic Pastor, Philipp Moroder

**Affiliations:** † Center for Musculoskeletal Surgery, Charité–Universitätsmedizin Berlin, Berlin, Germany; ‡ Schulthess Klinik, Zurich, Switzerland; § Klinikum Braunschweig, Braunschweig, Germany; Investigation performed at Charité Universitätsmedizin Berlin, Berlin, Germany

**Keywords:** constitutional posterior decentering, constitutional static posterior humeral decentering, muscle volume, preosteoarthritic deformity, static posterior shoulder instability

## Abstract

**Background:**

Constitutional static posterior humeral decentering (type C1 according to ABC Classification) has been recognized as a pre–osteoarthritic deformity that may lead to early-onset posterior decentering osteoarthritis at a young age. Therefore, it is important to identify possible associations of this pathologic shoulder condition to find more effective treatment options.

**Purpose:**

To perform a comprehensive analysis of all parameters reported to be associated with a C1 shoulder—including the osseous shoulder morphology, scapulothoracic orientation, and the muscle volume of the shoulder girdle in a single patient cohort.

**Study Design:**

Cross-sectional study; Level of evidence, 3.

**Methods:**

A retrospective, comparative study was conducted analyzing 17 C1 shoulders in 10 patients who underwent magnetic resonance imaging (MRI) with the complete depiction of the trunk from the base of the skull to the iliac crest, including both humeri. The mean age of the patients was 33.5 years, and all patients were men. To measure and compare the osseous shoulder morphology (glenoid version, glenoid offset, humeral torsion, anterior acromial coverage, posterior acromial coverage, posterior acromial height, and posterior acromial tilt) and scapulothoracic orientation (scapular protraction, scapular internal rotation, scapular upward rotation, scapular translation, scapular tilt, and thoracic kyphosis), these patients were matched 1 to 4 according their age, sex, and affected side with shoulder-healthy patients who had received positron emission tomography (PET)-computed tomography. To measure and compare the muscle volume of the shoulder girdle (subscapularis, infraspinatus/teres minor, supraspinatus, trapezius, deltoid, latissimus dorsi/teres major, pectoralis major, and pectoralis minor), patients were matched 1 to 2 with patients who had received PET-MRI. Patients with visible pathologies of the upper extremities were excluded.

**Results:**

The C1 group had a significantly higher glenoid retroversion, increased anterior glenoid offset, reduced humeral retrotorsion, increased anterior acromial coverage, reduced posterior acromial coverage, increased posterior acromial height, and increased posterior acromial tilt compared with controls (*P* < .05). Decreased humeral retrotorsion showed significant correlation with higher glenoid retroversion (*r* = −0.742; *P* < .001) and higher anterior glenoid offset (*r* = −0.757; *P* < .001). Significant differences were found regarding less scapular upward rotation, less scapular tilt, and less thoracic kyphosis in the C1 group (*P* < .05). The muscle volume of the trapezius and deltoid was significantly higher in the C1 group (*P* < .05).

**Conclusion:**

Patients with C1 shoulders differ from healthy controls regarding osseous scapular and humeral morphology, scapulothoracic orientation, and shoulder girdle muscle distribution. These differences may be crucial in understanding the delicate balance of glenohumeral centering.

The ABC classification separates acute (type A), dynamic (type B), and static (type C) posterior shoulder instability and further distinguishes static posterior shoulder instability into constitutional posterior decentering (C1) and acquired posterior decentering.^21^ C1 has been recognized as a pre–osteoarthritic deformity that may lead to early-onset posterior decentering osteoarthritis.^34^ At this time, C1 instability seems to be an irreversible progressive pathology, as suggested surgical options including open-wedge osteotomy,^5,23,35^ posterior bone grafting,^5^ or arthroscopic posterior articular coverage and shift^19^ have failed to permanently recenter the joint. Furthermore, patients are often affected at a young age. Therefore, it is important to identify possible associations of this pathologic shoulder condition to find more effective treatment options.

Differences in osseous glenoid morphology—in terms of increased glenoid retroversion and increased anterior glenoid offset—were identified as possible associations of the humeral static subluxation.^1,4,9^ Recently, a significant association between posterior shoulder instability and acromial anatomy was identified, showing that a high-riding and flat acromion may provide less osseous restraint against posterior humeral head translation.^15^ Although no information was provided for the severity of static humeral head subluxation of the included patients and patients with a glenoid dysplasia and retroversion of >15° were excluded in this study, acromial anatomy may also play a crucial role in patients with C1 shoulders.

Although our understanding of the pathoanatomy of scapular morphology in patients with C1 shoulders has improved, the humeral morphology in these patients has not yet been studied. Raniga et al^26^ found significant differences in patients with Walch type B shoulders compared with nonarthritic shoulders in terms of significantly less humeral retrotorsion. A reduced humeral retrotorsion may compensate for increased glenoid retroversion in patients with C1 shoulders.

It has been suggested that scapulothoracic orientation (eg, decreased scapular upward rotation) and a scapulohumeral muscle imbalance may also have a role in the pathogenesis of shoulder instability.^13,16,33^ The rotator cuff transverse force couple, which defines the balance between the forces of the subscapularis muscle and the infraspinatus and teres minor muscles, plays a crucial role in the normal kinematics of the shoulder, as it generates an antagonistic balance between anterior- and posterior-directed forces ensuring appropriate glenohumeral stability.^6,10,32^ Recent studies showed a balanced transverse force couple volume ratio in healthy individuals,^6,25^ whereas Mitterer et al^16^ showed an imbalanced transverse force couple with a comparatively large subscapularis muscle volume in young patients with static posterior humeral head subluxation without osteoarthritis. A greater anterior muscle volume may cause an increased posterior force vector, contributing to the static posterior humeral subluxation. In addition to rotator cuff musculature, the latissimus dorsi, deltoid, and pectoralis major were identified as capable of producing shoulder forces that may contribute to shoulder deformity after brachial plexus birth palsy.^2,3^ The role of these muscles in the cause of C1 has not yet been investigated.

This study aimed to perform a comprehensive analysis of all parameters currently reported to be associated with a C1 shoulder, including the osseous shoulder morphology, scapulothoracic orientation, and the muscle volume of the shoulder girdle in a single patient cohort. We hypothesized that patients with a C1 shoulder would show significant differences in osseous shoulder morphology, scapulothoracic orientation, and muscle volume compared with healthy controls.

## Methods

This was an observational, retrospective, cross-sectional, and comparative study. Patients with a C1 shoulder who were treated in our institution between February 2018 and March 2021 and underwent magnetic resonance imaging (MRI) in a supine position with arms at the side and elbows resting on the examination table with the complete depiction of the trunk from the base of the skull to the iliac crest, including both humeri, were included in this study. The exclusion criteria were concomitant rotator cuff tear and collective instability arthropathy index^20^ <2. None of the patients were excluded; thus, the study cohort comprised 17 shoulders in 10 patients. Seven patients had a bilateral involvement and the dominant hand was affected in the remaining 3 patients (right side in 2 patients and left side in 1 patient). The mean age of the patients was 33.5 ± 15 years, and all patients were men. The study protocol (application No. EA2/149/21) was reviewed and approved by the institutional review board (Charité Universitätsmedizin).

None of the patients had typical instability symptoms, but they reported pain, weakness, and clicking. The mean duration of symptoms was 28 ± 19 months. A traumatic event could not have been identified in any of the cases as a cause of the posterior instability. Also, 6 of 10 patients stated that they participated in sports activities routinely, including overhead sports in 4 of 6 patients. The remaining 2 patients reported they did weight lifting regularly.

To measure and compare the osseous shoulder morphology and scapulothoracic orientation, these patients were matched 1 to 4 according to their age (within 5 years), sex, weight, height, and affected side with patients from our institutional radiology database who had received positron emission tomography (PET)–computed tomography (CT) and met the following inclusion criteria: (1) supine position with the arms at the side and elbows resting on the examination table; (2) complete depiction of the trunk from the base of the skull to the iliac crest, including both humeri; (3) sufficient CT quality for 3-dimensional (3D) rendering; and (4) centered humeral head with no signs of glenohumeral osteoarthritis. Patients with visual pathologies of the upper extremities (eg, prostheses, tumor, fractures, or dysplasia) were excluded. CT imaging of the patients was performed as either low- or full-dose imaging (tube voltage 120/140 kV; automatic tube current modulation; primary slice thickness, 0.625 mm). A total of 68 healthy shoulders in 40 male control patients were identified and included in this study.

To measure and compare the muscle volume of the shoulder girdle, patients were matched 1 to 2 according to their age (within 5 years), sex, and affected side with patients from our institutional radiology database who had received PET-MRI scans and met the following inclusion criteria: (1) supine position with the arms at the side and elbows resting on the examination table; (2) complete depiction of the trunk from the base of the skull to the iliac crest, including both humeri; and (3) centered humeral head with no signs of glenohumeral osteoarthritis. Patients with visible pathologies of the upper extremities (eg, prostheses, tumor, fractures, or dysplasia) were excluded. MRI of the patients was performed for each shoulder at 3.0 T using phased-array body coils. Examination protocols included an axial T2-weighted turbo spin-echo sequence with a 3.00-mm slice thickness ranging from the base of the skull to the iliac crest, as well as a 3D SPACE (Sampling Perfection with Application optimized Contrast using different flip angle Evolution) sequence with a slice thickness of 1.00 mm centered around the scapula. A total of 34 healthy shoulders in 20 male control patients were identified and included in this study.

### Image Measurements

All measurements were performed with Visage software Version 7.1 (Visage Imaging).

### Osseous Shoulder Morphology and Scapulothoracic Orientation

**Figure 1.**
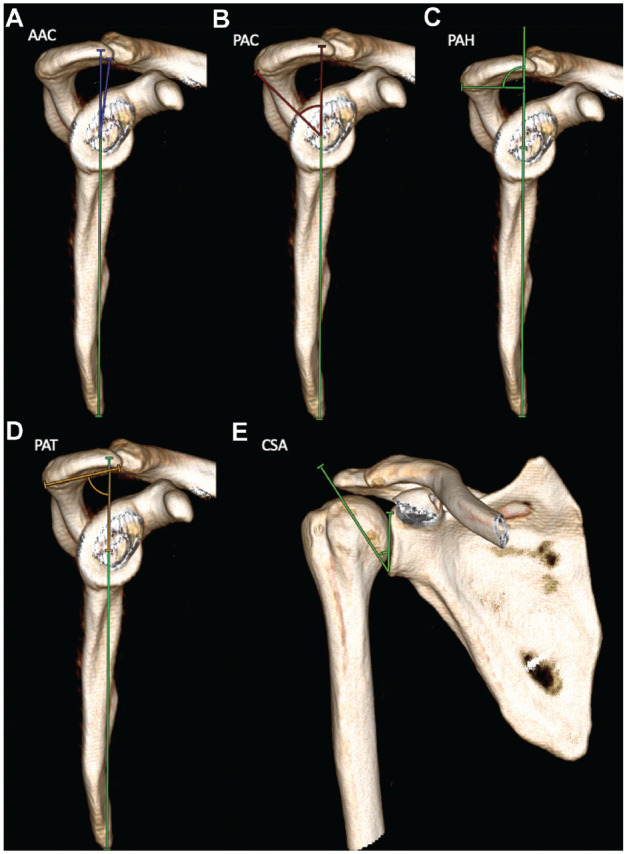
Measurements of the acromial morphology on 3-dimensional computed tomography models. (A) The AAC refers to an angle formed by the reference line connecting the inferior margin of the scapula with the center of the intersection of the small arms of the “Y” and a line drawn from the intersection of the small arms of the Y to the most anterior point of the inferior aspect of the acromion (blue angle). (B) The PAC refers to an angle formed by the same reference line and a line drawn from the intersection of the small arms of the Y to the most posterior point of the inferior aspect of the acromion (red angle). (C) The PAH is measured as the distance from the center of the intersection of the small arms of the Y to a perpendicular line that connects the reference line with the most posterior point of the inferior aspect of the acromion. (D) The PAT is measured as the angle formed by the reference line and a line connecting the most posterior point of the inferior aspect of the acromion to the most anterior point of the inferior aspect (brown angle).^15^ (E) The CSA is measured between a line connecting the inferior border to the superior border of the glenoid fossa and a line connecting the inferior border of the glenoid fossa to the most inferolateral point of the acromion.^17^ AAC, anterior acromial coverage; CSA, critical shoulder angle; PAC, posterior acromial coverage; PAH, posterior acromial height; PAT, posterior acromial tilt.

The glenoid version according to the method of Friedman et al,^8^ the humeral head subluxation using the glenohumeral and scapulohumeral subluxation indexes,^1^ and the glenoid offset according to the method of Akgün et al^1^ were measured on a 2-dimensional standardized axial imaging plane using a multiplanar reconstruction, as previously described. Glenoid dysplasia was defined as increased retroversion and posteroinferior osseous deformity.^36^ To measure the acromial morphology and scapulothoracic orientation, MRI data in patients with C1 shoulders and CT data in healthy controls were rendered into 3D models. Measurements of acromial morphology included anterior acromial coverage, posterior acromial coverage, posterior acromial height, and posterior acromial tilt according to Meyer et al,^15^ and critical shoulder angle^17^ (Figure 1). We conducted the measurements on 3D models of the scapula from a true sagittal view, where the coracoid process and scapular spine appeared as symmetric upper limbs of a “Y,” in contrast to Meyer at al, who conducted the measurements on a true lateral radiograph. Humeral torsion was measured as the angle between the epicondylar axis and the line perpendicular to the anatomic neck of the humeral head in the transversal plane.^14^

**Figure 2.**
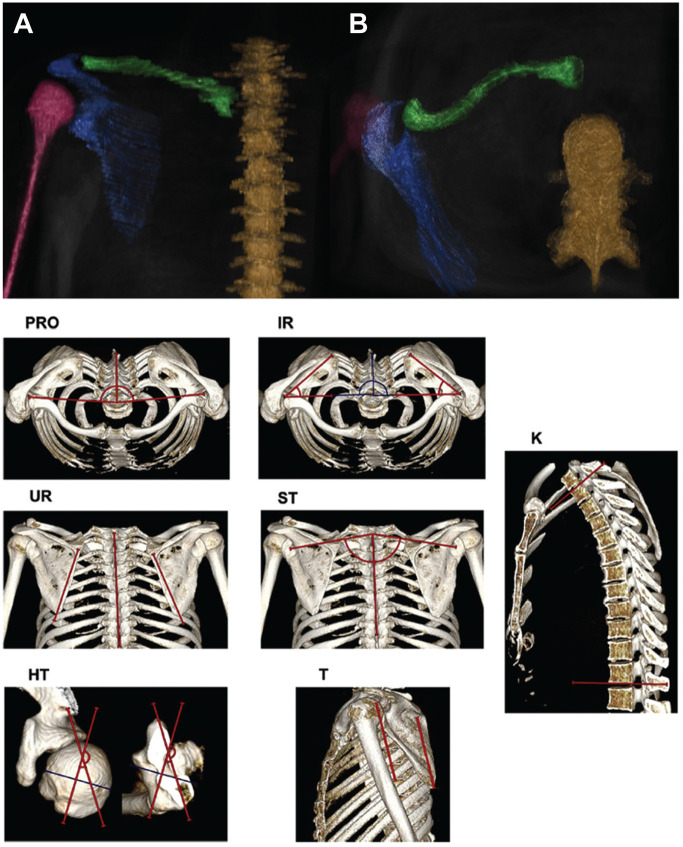
Three-dimensional (3D) models—(A) posterior and (B) axial views—were rendered from magnetic resonance imaging data in patients with C1 posterior shoulder instability to measure scapulothoracic orientation: spine (brown), clavicle (green), scapula (blue), and humerus (purple). Example measurements on 3D computed tomography models for scapular protraction (PRO), scapular internal rotation (IR), scapular upward rotation (UR), scapular translation (ST), global thoracic kyphosis (K), humeral torsion (HT), and scapular tilt (T) from the study of Moroder et al.^18^ C1, constitutional posterior decentering.

Scapular orientation measurements—including scapular protraction, scapular internal rotation, scapular upward rotation, scapular translation, scapular tilt, and thoracic kyphosis—were determined according to Park et al^24^ and Moroder et al^18^ (Figure 2).

### Muscles of the Shoulder Girdle

**Figure 3.**
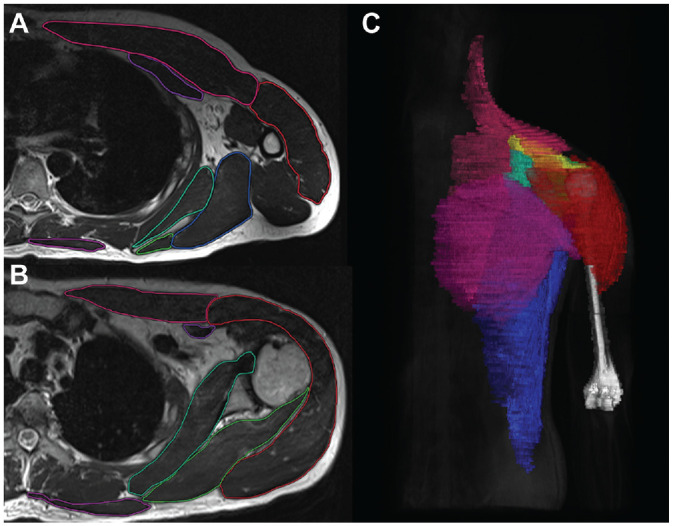
(A and B) Marking of shoulder girdle muscles on transverse slices. (C) Three-dimensional reconstructed model of measured muscles rendered from magnetic resonance imaging data. Deltoideus (red), pectoralis major (pink), subscapularis (turquoise), infraspinatus/teres minor (green), pectoralis minor (dark purple), latissimus/teres major (blue), trapezius (light purple), supraspinatus (yellow), and humerus (white).

Volumes of the following muscles were measured: trapezius, deltoid, supraspinatus, subscapularis, infraspinatus/teres minor, pectoralis major, pectoralis minor, and latissimus dorsi/teres major. Muscle contours were manually marked on every transverse slice for each measured muscle, and the muscle volume was calculated automatically from the software (Figure 3). Furthermore, the muscle volume ratio of the rotator cuff transverse force couple was calculated as previously described by Espinosa-Uribe et al.^6^

### Statistical Analysis

Two raters (H.G. and A.P.) conducted all the measurements independently at different time points except for the glenoid offset, glenoid version, and glenohumeral and scapulohumeral subluxation indices, which were already shown to have an almost perfect interobserver agreement in the literature.^1^ The intraclass correlation coefficient (ICC) with the 95% CI was calculated for all measurements. As recommended by Koo and Li,^12^ an ICC of <0.50 indicates poor reliability, 0.50 to 0.75 moderate reliability, 0.76 to 0.9 good reliability, and >0.90 excellent reliability. After reliability assessment, the values of both raters were averaged for further analysis. The Kolmogorov-Smirnov test was used to test for normal distribution. The 2-sample *t* test (for parametric distribution) or the Mann-Whitney *U* test (for nonparametric distribution) was used to compare continuous variables between groups. Correlation analyses were performed using the Pearson correlation coefficient. The results were given as means and standard deviations or as numbers and percentages. IBM SPSS Statistics 29.0 software (IBM) was employed for statistical analyses. *P* < .05 was considered significant.

## Results

No significant differences were found between the C1 group and the matched control group regarding age, sex, weight, and height (Table 1). All measurements showed good to excellent reliability between the 2 raters. ICCs are summarized in Table 2.

**Table 1:** Comparison of Descriptive Data of the C1 Group With the Control Group^*a*^

	C1 Group, n = 10	PET-CT Controls, n = 40	PET-MRI Controls, n = 20
Age, y	33.5 ± 15	32.1 ± 13.5	33.1 ± 15
Sex, male	10	40	20
Weight, kg	81.1 ± 11	79.5 ± 11	77.8 ± 12
Height, cm	180.6 ± 8	180.7 ± 8	179 ± 7

a Data are presented as mean ± SD or n. C1, constitutional posterior decentering; PET-CT, positron emission tomography–computed tomography; PET-MRI, positron emission tomography–magnetic resonance imaging.

**Table 2:** Calculated ICC for all Measurements^*a*^

	ICC	95% CI		Reliability
		Lower Bound	Upper Bound	
Humeral torsion Anterior acromial coverage Posterior acromial coverage Posterior acromial height Posterior acromial tilt Critical shoulder angle Protraction Scapular internal rotation Scapular upward rotation Scapular translation Scapular tilt Kyphosis Trapezius Deltoid Supraspinatus Subscapularis Infraspinatus/teres minor Pectoralis major Pectoralis minor Latissimus dorsi/teres major	0.873 0.966 0.951 0.911 0.965 0.931 0.941 0.899 0.960 0.827 0.893 0.889 0.863 0.975 0.904 0.907 0.940 0.962 0.904 0.956	0.601 0.948 0.925 0.862 0.936 0.855 0.908 0.815 0.917 0.537 0.838 0.799 0.127 0.955 0.824 0.582 0.684 0.710 0.802 0.901	0.943 0.978 0.968 0.942 0.979 0.963 0.962 0.940 0.978 0.916 0.930 0.938 0.956 0.986 0.947 0.965 0.978 0.988 0.950 0.978	Good Excellent Excellent Excellent Excellent Excellent Excellent Good Excellent Good Good Good Good Excellent Excellent Excellent Excellent Excellent Excellent Excellent

a ICC, intraclass correlation coefficient.

### Osseous Shoulder Morphology

The results of the measurements and comparisons between groups are summarized in Table 3. The C1 group had a significantly increased glenoid retroversion, increased scapulohumeral subluxation index, increased anterior glenoid offset, increased anterior acromial coverage, reduced posterior acromial coverage, increased posterior acromial height, increased posterior acromial tilt, reduced critical shoulder angle, and reduced humeral retrotorsion compared with healthy controls. Glenoid dysplasia was present in 9 of 17 shoulders (53%).

**Table 3:** Osseous Shoulder Morphology Measurement Comparison of the C1 Shoulders and Matched Controls^*a*^

	C1 Shoulders, n = 17	Controls, n = 68	*P*
Glenoid retroversion, deg	20.1 ± 10.7	5.8 ± 2.7	<.001
Glenohumeral subluxation index	0.56 ± 0.1	0.52 ± 0.03	.12
Scapulohumeral subluxation index	0.75 ± 0.1	0.57 ± 0.04	<.001
Anterior glenoid offset, mm	9.8 ± 4	3.9 ± 1	<.001
Anterior acromial coverage, deg	22 ± 6.7	14.1 ± 3.4	<.001
Posterior acromial coverage, deg	52.8 ± 11.1	63.2 ± 6.3	.002
Posterior acromial height, mm	21.9 ± 4.6	18.6 ± 4.7	.046
Posterior acromial tilt, deg	78.8 ± 5	69.8 ± 3.9	<.001
Critical shoulder angle, deg	27.4 ± 4.1	30.4 ± 1.7	.006
Humeral retrotorsion, deg	14.9 ± 11.2	34.4 ± 4.9	<.001

a Data are presented as mean ± SD. C1, constitutional posterior decentering.

### Scapulothoracic Orientation

The results of the measurements and comparisons between groups are summarized in Table 4. Significant differences were found between the 2 groups in terms of significantly less scapular upward rotation, less scapular tilt, and less thoracic kyphosis in the C1 group. Furthermore, a trend toward less scapular internal rotation in the C1 group was found, albeit statistically not significant.

**Table 4:** Scapulothoracic Orientation Measurement Comparison of the C1 Shoulders and Matched Controls^*a*^

	C1 shoulders, n = 17	Controls, n = 68	*P*
Protraction, deg	88.3 ± 4.6	90.2 ± 2.2	.16
Scapular internal rotation, deg	41.8 ± 6.3	45 ± 2.5	.09
Scapular upward rotation, deg	9.6 ± 3.6	12.2 ± 2.4	.03
Scapula translation, deg	78.8 ± 5	77.6 ± 3.6	.41
Scapular tilt, deg	7.9 ± 3.2	16.7 ± 3.5	<.001
Kyphosis, deg	24.9 ± 9.2	34.7 ± 4.7	.005

a Data are presented as mean ± SD. C1, constitutional posterior decentering.

### Muscle Volume

The results of the measurements and comparisons between groups are summarized in Table 5. The muscle volume of the trapezius and deltoid were significantly higher in the C1 group compared with the control group. The subscapularis showed a significantly higher muscle volume compared with the infraspinatus/teres minor in C1 shoulders as well as in healthy controls (194.3 ± 39 cm^3^ vs 177.6 ± 31.4 cm^3^, *P* = .04; and 194.3 ± 30.1 cm^3^ vs 181.7 ± 28 cm^3^, *P* = .002, respectively). No differences were found in the transverse force coupling of the rotator cuff between cohorts. No other differences in muscle volume were found.

**Table 5:** Muscle Measurement Comparison of the C1 Shoulders and Matched Controls^*a*^

	C1 shoulders, n = 17	Controls, n = 34	*P*
Trapezius, cm^3^	248 ± 42.9	199 ± 40.1	.005
Deltoid, cm^3^	500.3 ± 79.5	386.8 ± 49.5	.0001
Supraspinatus, cm^3^	62.6 ± 13.8	62 ± 8.6	.89
Subscapularis, cm^3^	194.3 ± 39	194.3 ± 30.1	.99
Infraspinatus/teres minor, cm^3^	177.6 ± 31.4	181.7 ± 28	.69
Muscle volume ratio of RC-TFC	1.1 ± 0.2	1.07 ± 0.1	.41
Pectoralis major, cm^3^	423.8 ± 55	387.7 ± 114.3	.27
Pectoralis minor, cm^3^	50.5 ± 7.1	48.2 ± 9.7	.51
Latissimus dorsi/teres major, cm^3^	490 ± 73.5	472.7 ± 110.9	.57

a Data are presented as mean ± SD. C1, constitutional posterior decentering; RC-TFC, rotator cuff transverse force couple.

### Correlations

A decreased humeral retrotorsion showed a significant correlation with a higher glenoid retroversion (*r* = −0.742; *P* < .001) and a higher anterior glenoid offset (*r* = −0.757; *P* < .001).

A higher glenoid retroversion showed a significant correlation with a higher scapulohumeral subluxation index (*r* = 0.82; *P* < .001) and a higher glenoid anterior offset (*r* = 0.723; *P* < .001).

## Discussion

This study comprehensively analyzed all parameters currently under investigation that might be associated with a C1 shoulder. We found significant differences in osseous shoulder morphology, scapulothoracic orientation, and muscle volume of the shoulder girdle in patients with C1 shoulders compared with healthy controls.

In addition to previously described constitutional bony abnormalities—including increased glenoid retroversion and increased anterior glenoid offset^1,4,34^—this study also showed a significantly different osseous acromial morphology in patients with C1 shoulders compared with healthy controls. Our results are almost in accordance with the findings of Meyer et al^15^ who first described the association between posterior shoulder instability and a higher and more horizontally oriented acromion. Although Meyer et al^15^ did not provide any information about the severity of the static humeral head subluxation of the included patients and even excluded patients with glenoid dysplasia and retroversion of >15°, the theory that a high-riding and flat acromion may provide less osseous restraint against posterior humeral head translation also seems to be applicable for patients with C1 shoulders. Interestingly, patients with C1 shoulders in our cohort had significantly more anterior acromial coverage than healthy controls, which may promote static posterior instability. Given that trauma does not play any role in the development of a C1 shoulder, the lack of a posterior osseous restraint created by the posterior aspect of the acromion may lead to overloading of the posterior capsular structures, resulting in static posterior decentering.

In addition to the differences in scapular morphology, patients with C1 shoulders showed significantly less humeral retrotorsion compared with healthy controls. We also found a linear correlation between decreased humeral retrotorsion, increased glenoid retroversion, and increased anterior glenoid offset. Recently, Raniga et al^26^ described significantly less humeral retrotorsion in patients with a Walch type B humerus compared with healthy controls and stated that the significantly reduced humeral retrotorsion may be an etiologic factor that may lead to the posteroinferior glenoid wear and its evolution. It is unknown whether the reduced humeral retrotorsion is the reason or consequence of increased glenoid retroversion and increased anterior glenoid offset; nonetheless, there seems to be a delicate balance of both. This balance may be the reason that the humeral head, despite its misalignment with the scapula and the rotator cuff action lines, is indeed perfectly centered to the glenoid fossa, meaning that both humeral torsion and glenoid version, as well as glenoid offset, compensate each other to achieve a well-centered glenohumeral joint. This is also confirmed by the results of our study, which show a well-centered glenohumeral joint in C1 shoulders with no difference from healthy controls. This finding also has potential surgical implications. Although open-wedge osteotomy alone cannot restore the shoulder stability,^5,23,35^ its combination with a humeral rotational osteotomy aiming to increase the humeral retrotorsion may be an effective surgical treatment.

There is a growing body of literature associating abnormal scapular kinematics with shoulder pathologies, including glenohumeral instability. Despite a wide range of variation in study samples and methodologies, various studies identified less scapular upward rotation, greater scapular internal rotation, and reduced anterior tilt/increased posterior tilt in patients with glenohumeral instability during elevation of the arm in the scapular plane.^13,22,29,31^ Although the scapular orientation angles measured in this study were obtained in a supine and static position, we found significant differences between C1 shoulders and healthy controls in terms of reduced scapular upward rotation and reduced anterior tilt, consistent with the literature. Identifying the exact mechanism causing abnormal kinematics in patients with a C1 shoulder is difficult. On one hand, this disruption in normal scapular movement may be a response to a painful shoulder condition, which explains why scapular dyskinesia is evident in other shoulder pathologies causing pain, such as impingement.^11^ On the other hand, scapular malposition may cause biomechanical alterations; it has been suggested that the altered position of a downwardly rotated scapula causes a decrease in inferior stability.^22^ Although it is not known whether the aberration of scapular orientation seen in our study cohort compared with healthy controls was the cause or the result of the static humeral decentering, the kinematic deviations found are believed to be detrimental with regard to glenohumeral stability.^22^

The previously described larger subscapularis muscle volume compared with infraspinatus/teres minor was also confirmed in our study—both in C1 shoulders and in healthy controls.^16^ The main limitation of the study by Mitterer et al^16^ is the lack of a healthy control group. Additionally, although 2 studies^6,25^ showed no significant differences between muscle volume of the subscapularis and infraspinatus/teres minor in healthy individuals, age differences and sample size can indeed make a direct comparison of different cohorts difficult. In the study of Piepers et al,^25^ only 27 shoulders in 21 patients with a mean age of 57 years were analyzed. As shown in the study of Espinosa-Uribe et al,^6^ muscle volume tends to correlate with age, with the highest volumes occurring between 18 and 40 years, with a rotator cuff transverse force couple of 1.05 for men and even 1.07 for women between ages 18 and 29 years. The rotator cuff transverse force couple in our perfectly matched cohort is also comparable with the results of Espinosa-Uribe et al, suggesting that a slight difference between subscapularis and infraspinatus/teres minor volumes can be physiological at younger ages. The detected significant differences in muscle volumes of the trapezius and deltoid are described for the first time in the literature for this patient population and cannot be explained clearly. Additional studies are needed to determine the causal relationship of these muscle changes to static humeral decentering and differences in scapulothoracic orientation.

This study has several limitations. Although standardized imaging for all the measurements was used, small variations in alignment can significantly affect the measurements. To reduce this limitation, 2 raters conducted the measurements independently, showing almost perfect agreement in all measurements. The supine position of the patients with their arms at the side without any standardized arm rotation may also alter our results, especially scapulothoracic orientation by shifting the scapula toward retraction and attenuating kyphosis. Although utilization of 2 different modalities (MRI in patients with C1 shoulders and CT in control patients to measure osseous shoulder girdle anatomy) may seem to alter the comparability of our results, 3D-MRIs are described in the literature as reliable and as accurate as conventional CT in the diagnosis and evaluation of osseous pathologies of the shoulder girdle.^7,27,28,30^ The small sample size of the C1 group may underpower our results. Nonetheless, the differences were substantial enough to reach statistical significance even with a small sample size. Patients undergoing PET-CTs or PET-MRIs are much more likely to have systemic disease and may have, therefore, despite matching according to weight and height, different muscular anatomy compared with patients with C1 shoulders, which may alter our results.

## Conclusion

Patients with a C1 shoulder do differ from healthy controls in terms of osseous scapular and humeral morphology, scapulothoracic orientation, and shoulder girdle muscle distribution. These differences may play a crucial role in understanding the delicate balance of glenohumeral centering.
